# Online Adaptive Radiotherapy for Planning Target Volume (PTV) Reduction in Gastric Mucosa-Associated Lymphoid Tissue (MALT) Lymphoma

**DOI:** 10.7759/cureus.68919

**Published:** 2024-09-08

**Authors:** Thaïs Tison, David Dechambre, Julien Pierrard, Louise Everard, Xavier Geets

**Affiliations:** 1 Radiation Oncology, Cliniques Universitaires Saint-Luc, Brussels, BEL; 2 Medical Physics, Cliniques Universitaires Saint-Luc, Brussels, BEL; 3 Medical Physics, Center of Molecular Imaging, Radiotherapy and Oncology (MIRO) Institut de Recherche Experimentale et Clinique (IREC) Université Catholique de Louvain (UCLouvain), Brussels, BEL

**Keywords:** gastric lymphoma, interfraction motion, online adaptive radiotherapy, planning target volume, real-time intrafraction motion

## Abstract

Online adaptive radiotherapy (oART) uses daily imaging to identify changes in the patient's anatomy and generate a new treatment plan adapted to these changes, and it can be used for treating gastric mucosa-associated lymphoid tissue (MALT) lymphomas. This study aimed to determine the intrafraction motion and planning target volume (PTV) margins required for an oART workflow on a cone beam computed tomography (CBCT)-based dedicated system (Ethos®, Varian Medical Systems, Palo Alto, California, United States) and investigate the potential benefits for patients compared with a non-adaptive workflow. Involving three patients treated for gastric MALT lymphoma with the oART under breath-hold (BH) technique, the study determined a PTV margin for adaptive treatment using CBCT scans performed at the beginning and just before treating the patients for 34 fractions. Different PTVs were made by isotropically extending the clinical target volume (CTV) contoured on the first CBCT (CTV1) at intervals of 1 mm to evaluate intrafraction gastric motion, with the expansion covering the contoured CTV on the second CBCT (CTV2) quantifying the intrafraction motion (adaptive treatment) and the expansion from the CTV delineated on the planning scanner (CTVplanning) that could cover both CTV1 and CTV2 defining the interfraction motion (non-adaptive treatment). PTV margins were then determined as the extension of the CTV allowing coverage of 95% of its volume in 90% of fractions, and the dosimetric impact on dose constraints between an adaptive plan and a non-adaptive plan based on the predetermined margins was evaluated. A total of 68 CBCTs were analyzed, revealing that the PTV margin for oART was 4 mm, while for non-adaptive treatment it was 12 mm, with an average time elapsed between CBCT1 and CBCT2 of 11.62 minutes and no correlation between inter-CBCT timing and PTV margins (Pearson R-coefficient=0.10). All dosimetric constraints were met in both adaptive and non-adaptive plans, but the adaptive plan allowed for reduced organ-at-risk (OAR) doses in each patient. The study concluded that oART could reduce PTV margins in the treatment of gastric MALT lymphoma, especially with a BH strategy, impacting OAR dosimetry, though more prospective studies are required to validate these findings and determine their clinical impact on patients.

## Introduction

Gastric mucosa-associated lymphoid tissue (MALT) lymphomas are rare and account for 4% of all non-Hodgkin lymphomas [1,2]. They are associated with *Helicobacter pylori* (HP) infection in 90% of cases [3,4]. Initial treatment involves HP eradication, resulting in remission in approximately 75% of cases [5]. In case of failure, radiotherapy is an option, achieving remission in 96-100% of cases [2]. Due to the high radiosensitivity of gastric lymphoma, low radiation doses are sufficient. Typically, treatment covers the entire stomach at a dose of 20-36 Gy delivered in daily fractions ranging from 1.5 to 2 Gy [2].

Radiotherapy for gastric lymphoma is challenging due to its subdiaphragmatic location, making it susceptible to respiratory motion. Several studies have investigated the importance of using motion management techniques during radiotherapy, with breath-hold (BH) being the most commonly used. By freezing the respiratory-related motion, BH allows to reduce planning target volume (PTV) margins while maintaining adequate clinical target volume (CTV) coverage [6,7]. Also, the stomach is a deformable organ, with significant volume and shape changes. For this reason, the use of conservative and large PTV is still recommended.

Online adaptive radiotherapy (oART), which allows to disregard interfraction anatomical changes, could potentially help to reduce PTV margins, minimizing unnecessary irradiation of healthy tissues surrounding the target volume. The primary objective of this case study is to determine how far PTV margins could be reduced with oART compared to conventional non-adaptive radiotherapy while maintaining adequate CTV coverage. Then, we investigate whether such margin reduction translated into better dose sparing of surrounding organs-at-risk (OARs).

## Case presentation

We present here three patients who underwent oART at our academic hospital for gastric MALT lymphoma from July 2023 to December 2023. This study adhered to the Declaration of Helsinki and received approval from the Ethics Committee of Cliniques Universitaires Saint-Luc.

Planning computed tomography (planning CT) and radiotherapy treatments were performed in supine position, with arms raised, and without intravenous contrast administration. BH was used to mitigate the breathing motion, using surface image guidance (VisionRT® (Version 1.0, Vision RT Ltd., Dove House, London, United Kingdom) during planning CT and Identify® (Version 2.3.1, Varian, a Siemens Healthineers Company, Palo Alto, California, United States) during treatment), and patients were asked to fasten for at least three hours before each session to reduce stomach interfraction changes. Target volumes and OARs were contoured using RayStation® (Version12A, RaySearch Laboratories, Stockholm, Sweden), following international guidelines [8]. The CTV encompassed the entire stomach. The PTV incorporated a predetermined anisotropic margin (7 mm left-right, 8 mm superior-inferior, and 9 mm anterior-posterior) specifically used in our institution. Treatment planning was performed using the Ethos® planning system (Version 2.01.00, Varian). The radiotherapy treatment consisted of 12 daily oART fractions of 2 Gy (total dose of 24 Gy), delivered using 9-12 equidistant intensity-modulated beams on the Ethos® linear accelerator (Version 4.0, Varian).

During treatment sessions, BHs were performed during cone beam computed tomography (CBCT) acquisition and beam delivery. A first CBCT (CBCT1) scan was performed prior to adaptation and used for the automated delineation of target volumes and OARs of interest (stomach (CTV1), heart, liver, bowel, right lung, and left lung). Target volumes were propagated using a surface-guided deformable registration between the planning CT and the CBCT1 based on the daily anatomy of OARs called "influencers." These volumes were reviewed and, if necessary, corrected by a radiation oncologist. Then, the Ethos® software proposed two plans: the adapted plan was reoptimized according to the new anatomy and the clinical goals set at planning, while the scheduled plan corresponded to the reference plan recomputed on the actual anatomy, without adaptation. The plan that best matched the clinical goals was finally selected for treatment delivery. Prior to treatment delivery, a second CBCT (CBCT2) was performed to assess the intrafraction anatomical changes during the adaptation time. In case of positional deviation between CBCT1 and CBCT2, a local matching using translational shift was performed to ensure optimal coverage of the stomach by the PTV margin.

After all treatments were completed, Ethos® plans and CBCTs from each session were imported into Eclipse® (Version 16.01.10, Varian). Patient characteristics are described in Table 1. All except two CBCTs were successfully imported and analyzed, for a total of 68 CBCTs.

**Table 1:** Patient characteristics CBCT: cone beam computed tomography; F: female; M: male

	Patient 1	Patient 2	Patient 3
Gender	F	F	M
Age (years)	48	52	37
Number of CBCTs	22	24	22

**Figure 1:**
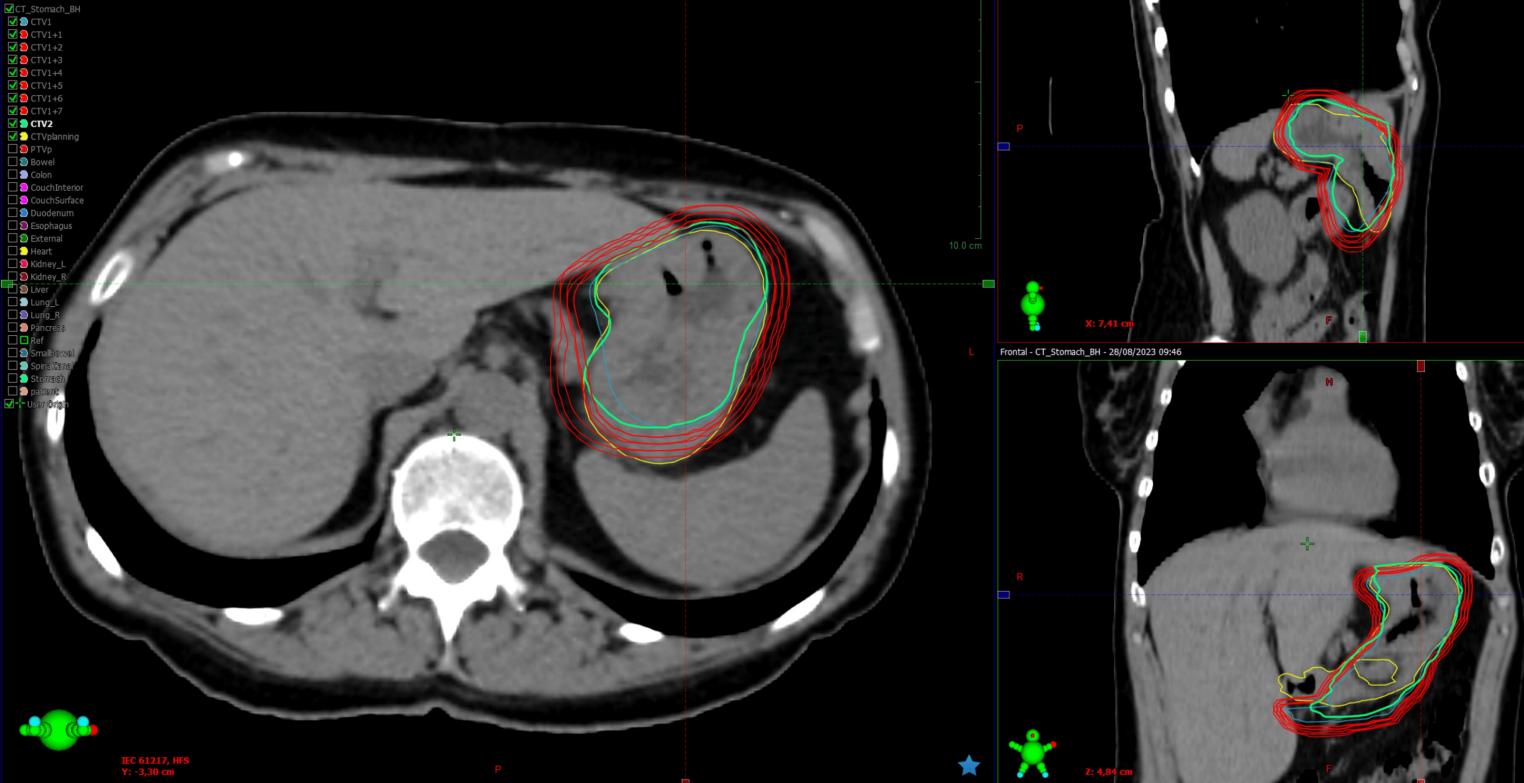
Example of CTV1 extensions used to determine PTV margins for oART in patient 1 (fraction number 1) PTV: planning target volume; oART: online adaptive radiotherapy; yellow curve: CTVplanning; blue curve: CTV1; green curve: CTV2; red curves: 1 mm isotropic extensions from CTV1

**Figure 2:**
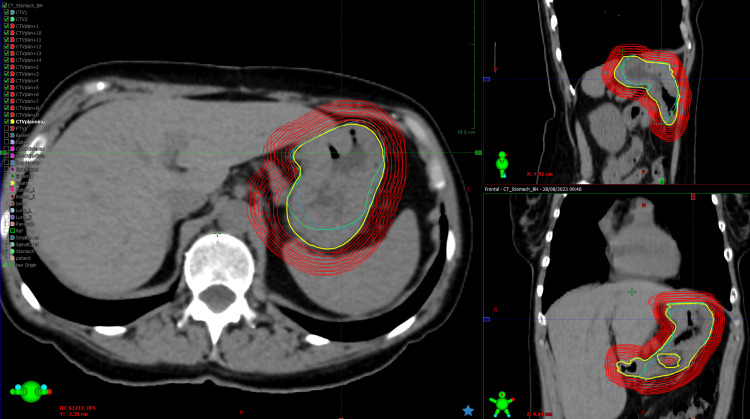
Example of CTVplanning extensions used to determine PTV margins for conventional non-adaptive treatment in patient 1 (fraction number 1) PTV: planning target volume; yellow curve: CTVplanning; blue curve: CTV1; green curve: CTV2; red curves: 1 mm isotropic extensions from CTVplanning

All CBCT2 were rigidly registered with the fraction-wise CBCT1, based on the stomach and using only translations. The stomach on the CBCT2 (CTV2) was manually delineated and mapped on the CBCT1 geometry. In order to determine the minimal PTV margin suitable for adaptive treatment, a 1 mm isotropic increment of the CTV1 was created until 95% of the CTV2 was covered by the PTV. To simulate conventional non-adaptive treatment, all CBCTs were rigidly registered to the planning CT, and both CTV1 and CTV2 were mapped onto its geometry. A similar margin expansion process was applied to determine the minimal suitable margin, applied onto the CTV delineated on planning CT (CTVplanning) and covering 95% of the two CTVs. Visual examples of those processes are shown in Figure 1 and Figure 2. To be consistent with the statistical nature of the margin formulae of the PTV, the 90th percentile of the margin expansion values were selected. 

**Figure 3:**
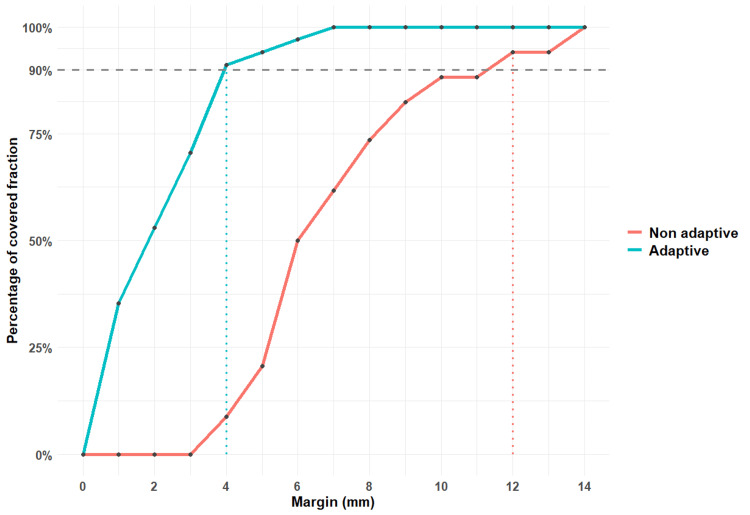
Percentage of fractions covered as a function of the PTV margin used for all three patients PTV: planning target volume; blue curve: margin for adaptive treatment; red curve: margin for non-adaptive treatment

For oART, a 4 mm isotropic PTV margin from CTV1 was sufficient to cover 95% of CTV2 in 90% of fractions. In contrast, for the non-adaptive treatment, a 12 mm isotropic PTV margin from CTVsim was needed to cover 95% of both CTV1 and CTV2 in 90% of fractions. The comparison of those margins between the two treatment methods is represented in Figure 3.

**Figure 4:**
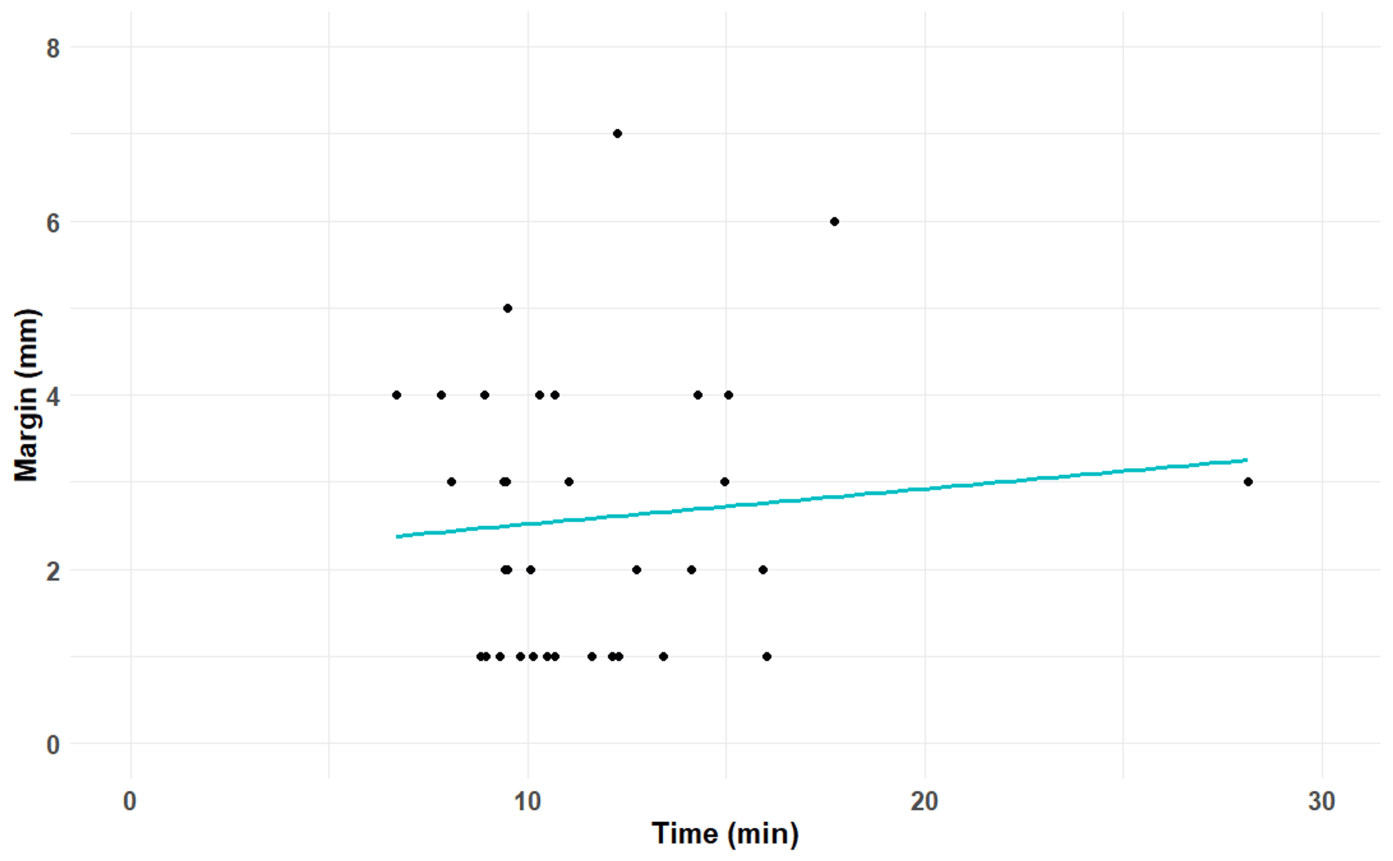
Correlation between inter-CBCT timing and PTV margins for intrafraction motion. CBCT: cone beam computed tomography; PTV: planning target volume

The time between acquiring CBCT1 and CBCT2 (inter-CBCT timing) was also recorded. The average time elapsed between CBCT1 and CBCT2 was 11.62 minutes (6.7-28.13), and no correlation was found between inter-CBCT timing and margin for adaptive strategy (Pearson R-coefficient=0.10, p=0.58) (Figure 4).

To estimate the dosimetric impact on OARs, two treatment plans were generated on the Ethos® planning system, using the PTV margins previously computed from either adaptive or non-adaptive treatments. To mitigate planning biases, both plans were generated with the same number of beams. PTVs and relevant dose metrics for OARs were recorded and compared between the two plans.

The mean PTV volume for adaptive treatment was 335 cc (297.5-402.8), compared to 664 cc (601-778.4) for non-adaptive treatment.

Dose metrics for TV and OARs are summarized in Table 2. All dose constraints were met, whether for the adaptive or non-adaptive plan. However, as expected by PTV reduction, the adaptive plan systematically reduced the dose to the relevant OARs.

**Table 2:** Dosimetric differences between a plan created with a 4 mm margin (adaptive plan) and a 12 mm margin (non-adaptive plan) A: adaptive; NA: non-adaptive; CTV: clinical target volume; KidneyL: kidney left; Const.: dose constraints; Δ: average difference between adaptive and non-adaptive data

	Patient	Plan	CTV V95% (%)	CTV V100% (%)	Liver Dmean (Gy)	Liver V20Gy (%)	Liver V15Gy (%)	KidneyL Dmean (Gy)	KidneyL V20Gy (%)	Heart Dmean (Gy)
	1	A	100	99.7	6.9	4.3	8.7	4.5	0	0.4
		NA	100	99.7	9.6	9.8	18.6	4.7	0	0.7
	2	A	100	99.9	7.2	3.8	8	2.0	0	1.1
		NA	100	99.6	7.9	7.8	11.9	2.6	0	2.4
	3	A	100	99.9	7.6	3.8	9.3	2.9	0	1.3
		NA	100	99.6	8.8	10.7	17.7	3.6	0	2.6
Const.	-	-	-	-	<26	<49	<59	<16.2	<30	≤5
Δ	-	-	-	-	1.6	5.5	7.4	0.5	0	0.9

## Discussion

This case study allows to assess the minimal PTV margins required for the radiotherapy treatment of gastric MALT lymphomas in the context of adaptive treatment (considering only intrafraction motion) and non-adaptive treatment (considering both inter- and intrafraction motion). To estimate the impact of residual intrafraction deformation, we used the CBCTs acquired during Ethos® adaptive sessions at two time points: prior to adaptation (CBCT1) and prior to delivery (CBCT2). In this cohort, the BH technique was used to mitigate breathing motion. We showed that adaptive treatment allowed to significantly reduce PTV margin from 12 to 4 mm, when only intrafraction motion was considered. This led to a mean PTV reduction of about 50%. Moreover, these data suggest that the 10 mm PTV margin commonly used in clinical practice for BH treatments, and the anisotropic margins specifically used in our institution, might be insufficient for non-adaptive treatment [6,7].

Reducing PTV margin and volume translated into substantial gains in irradiated volume at the prescribed dose and dose reduction to OARs for adaptive treatment. Given the excellent local control and long-term survival after radiotherapy for gastric lymphomas, minimizing treatment-related side effects is crucial. It has already been demonstrated that reducing the treatment field improves acute and late side effects and survival in patients undergoing this type of treatment [9].

Previous studies assessing intrafraction motion of the stomach often reported margins larger than 4 mm, but primarily focused on respiratory-related motion, which has been mitigated with the BH techniques in our cohort [10-12]. Similarly, studies on interfraction motion also suggested the need for larger margins but were conducted without this technique [11,13-15].

Hirose et al. recently published a study aiming to determine the PTV margins for adaptive and non-adaptive treatments in patients undergoing BH radiotherapy for gastric MALT lymphomas. They concluded that a margin of 14 mm should be applied for adaptive treatment, while a 25 mm margin is recommended for non-adaptive treatment [16]. The difference between their findings and those reported in our study is likely due to the different registration procedures, which were based on bony anatomy, whereas we performed a locally optimized registration on the stomach (CTV). Similar studies have been conducted for other tumor sites. For instance, Byrne et al. investigated appropriate PTV margins for adaptive prostate cancer treatment by registering the prostate (CTV) between CBCT2 and CBCT1. Their study found smaller margins than those commonly used for non-adaptive treatment and resulted in improved CTV coverage with oART [17].

In this study, we did not find a correlation between the time elapsed between CBCT1 and CBCT2 and adaptive PTV margins that are related to intrafraction motion.

This study has some limitations, including the retrospective nature and the small number of patients included. However, the analysis relied on a total number of 34 fractions and 68 CBCTs. We focused on uncertainties related to stomach deformation and did not quantify those associated with other factors, such as reproducibility of BH, delineation uncertainties, or positioning. Regarding BH reproducibility, existing studies yield conflicting results. Some demonstrate that uncertainties of 2 mm or less can be achieved and suggest greater variability in interfraction than intrafraction motion [18-21], while others report intra-BH displacements of up to 1 cm [22-24].

## Conclusions

This study presents a series of patients treated for gastric MALT lymphoma with oART in our institution. The aim of this study was to determine the PTV margins that could be used for this kind of treatment, when considering only the residual intrafraction deformation estimated from CBCT, and to compare it with PTV margins that should be used for conventional non-adaptive treatment, considering inter- and intrafraction motion. We found that a 4 mm PTV margin could be sufficient to cover 95% of the CTV in 90% of fractions for oART with image verification, whereas a 12 mm PTV margin would be required for non-adaptive treatment, resulting in an absolute difference of 8 mm. This PTV reduction between oART and conventional treatment also allows a dose reduction to OARs.

These findings suggest a clear benefit of oART in reducing treatment fields and doses to the patient and to OARs surrounding the target volume. Prospective studies are necessary to confirm these hypotheses and assess the clinical impact of these benefits. The doses used in the treatment of gastric MALT lymphoma are low, and the clinical impact could be not significant, but our findings exemplify the utility of adaptive treatment for organs with significant deformation potential.
